# Sub-seasonal Levee Deformation Observed Using Satellite Radar Interferometry to Enhance Flood Protection

**DOI:** 10.1038/s41598-019-39474-x

**Published:** 2019-02-25

**Authors:** Işıl E. Özer, Stephan J. H. Rikkert, Freek J. van Leijen, Sebastiaan N. Jonkman, Ramon F. Hanssen

**Affiliations:** Delft University of Technology, Faculty of Civil Engineering and Geosciences, Stevinweg 1, 2628 CN Delft, The Netherlands

## Abstract

Levees are critical in providing protection against catastrophic flood events, and thus require continuous monitoring. Current levee inspection methods rely on limited information obtained by visual inspection, resulting in infrequent, localized, mostly qualitative and subjective assessments. This hampers the timely detection of problematic locations and the assessment of levee safety in general. Satellite radar interferometry yields weekly observations of levee conditions with high precision which complement current inspection methods. Here we show that levees are susceptible to short-term swelling and shrinkage associated with meteorological conditions, and assess how deformations can be related to the geohydrological properties and the safety of the levee. Our findings allow to understand the sub-seasonal behaviour of the levee in greater detail and to predict swelling and shrinkage due to variation of the loading conditions. This will improve the detection of anomalous levee responses which contributes to the development of reliable early warning methods using continuous deformation monitoring.

## Introduction

Failures of flood defence systems often lead to significant human, economic, social, and environmental losses, with the risk being highest in densely populated areas^1,2^. Earthen levees form a large part of the existing flood defence systems. Depending on the natural or man-induced driving mechanisms, levees can fail due to hydraulic failures (e.g., overtopping) and/or geotechnical failures (e.g., instability, erosion)^3,4^. Consequently, the monitoring of these levees is critical in achieving safety standards and avoiding catastrophic flooding events. Being able to identify if, where, and when a failure would suddenly occur is an important aspect to consider with respect to safety. However, current conventional levee inspection methods mostly rely on expert observers^3,5^, which result in infrequent, qualitative and labour intensive assessments^6–8^. During these inspections, the integrity of the structures is assessed using visual inspection parameters, in order to check the presence of any damage, cracks, seepage, animal burrows, or irregular vegetation on the levees^9^. Thus, visual inspection by expert’s judgement is not effective in detecting small (mm- to cm-level) and gradual changes in the structural behaviour of the levee^10^, which may be indicative of an imminent failure. Common remote sensing (e.g. LiDAR, thermal infrared) and *in-situ* monitoring methods (e.g. levelling, GNSS, creep/strain meters) are also costly and time-consuming, and are therefore usually only applied to locations considered to be at high risk by a visual inspection. Hence, there is a need for innovative and cost-effective complementary techniques, especially in countries with an extensive amount of flood defence infrastructure, such as the Netherlands, China, UK, and US among many others. These techniques could be most beneficial if they contribute to detecting which locations are most prone to sudden failures and estimating far in advance whether a levee would fail. This can be done by analysing the response of a levee under normal loading conditions in order to identify locations at risk of failing during extreme loading conditions, such as storm, high river discharge or drought.

The deformation behaviour of a levee can be divided into two main categories. Long-term, interannual deformation, such as the subsidence of the levee occurring over a period of years, mainly depends on the type and composition of the soil and can be considered irreversible. Apart from this, levees show short-term, sub-seasonal deformation, e.g. due to changing water levels, precipitation, and temperature, occurring over periods of days to weeks depending on the soil and loading conditions. The change in soil volume due to variations in soil moisture content is denoted as the swelling and shrinking behaviour of the soil^11^, which has been studied for different soil types, such as clay^12,13^, peat^14–16^ and others^17–19^.

Understanding and monitoring levee behaviour over an extended area, however, requires the availability of frequent deformation data with high resolution^4^, which are not provided by current inspection methods. In recent years, satellite radar interferometry, also known as Interferometric Synthetic Aperture Radar (InSAR)^20,21^, has become an efficient tool to monitor the surface displacements, referred to as deformations in the rest of the paper. The technique provides millions of observations with meter-level spatial resolution and millimetre-level measurement precision at reasonably low costs^2,4^. SAR satellites allow day and night monitoring of the Earth’s surface in all weather conditions, with up to a sub-weekly repeat interval. Persistent Scatterer InSAR (PS-InSAR) is a processing technique to estimate deformation time-series of points in the interferometric data stack with a coherent reflection over time^20^. It has been successfully applied on urban areas^22^, railways^23^, dams^24^, highways^25^, landslides^26^, tectonic movements^27^, and land subsidence^28^. Applications on levees have been limited to interannual deformations to monitor subsidence of the levees^4,7,29–31^. However, as most geotechnical failure mechanisms are related to dynamic levee responses to changes in loading conditions happening on a time scale of days to weeks^4^, a better understanding of the short-term behaviour of levees is required. Understanding the levee responses in normal conditions can then help to detect or predict the levee response to more extreme conditions, which would increase our capability of detecting anomalies that could identify unsafe situations. In this study, we assess how sub-seasonal patterns due to swelling and shrinkage can be identified from continuous levee deformation observations obtained with PS-InSAR, and we analyse how these patterns are related to meteorological variations and levee safety. By determining whether the observed deformation is in line with the response predicted from loading conditions experienced by levees and relating it to geohydrological properties of levees, it would become possible to identify problematic locations and apply the appropriate countermeasures. Here we focus on earthen canal levees in Delft, located in the Netherlands, where almost 12 million people live in flood prone areas, and reliable flood defences are essential to prevent catastrophic flood events^32^.

## Modelling the Swelling and Shrinkage Behaviour of Levees

Swelling and shrinkage result from changes in the pore water pressures inside the levee, which are due to variations in hydrological loading conditions. When the soil saturates, the pore water pressure in the soil increases, reducing effective stresses in the soil matrix and results in swelling. In turn, a reduction in pore pressures due to drying leads to shrinkage of the soil^33^.

The swelling and shrinkage behaviour of the soil is especially relevant for the safety of the canal levees. Water levels in these canals are fairly constant and typically exceed surface levels of adjacent polders, posing a continuous flooding threat to the hinterland, even under normal conditions. These canal levees were often built centuries ago and strengthened several times using local peat and clay, amply available materials in the Netherlands^34^. Changes in precipitation and temperature can lead to significant swelling and shrinkage behaviour of these types of soil. In addition, cracks can form when the levee dries and the soil shrinks. Through these cracks water can enter the levee, reducing the soil strength. Another concern is that other materials or debris may enter the crack, preventing it from closing properly when the soil returns to a wet condition again^3^. Hence, the resulting changes in the geohydrology of the levee, which is loaded by a fairly constant water level, can directly lead to instability and failure. Many failures of the canal levees in the Netherlands have been recorded due to the heavy rainfall, e.g. a failure close to Wilnis in 1874^35^ or extreme warm and dry weather, such as failures in Zoetermeer in 1947, Oostzaan near Amsterdam in 1990, Bleiswijk in 1990^36^ and near Wilnis in 2003. Hence, extreme conditions, i.e. too dry (high temperature and low precipitation) or highly saturated soil (mainly heavy precipitation with low temperatures) cause a reduction in the soil strength of the levee, which can lead to a failure.

**Figure 1:**
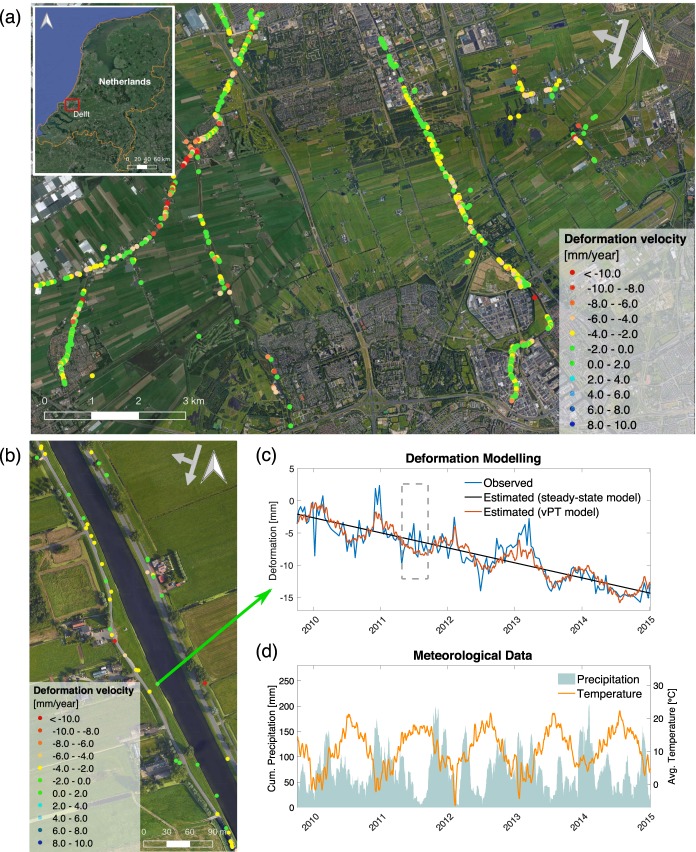
Analysis of deformation behaviour of the levees in Delft, the Netherlands. (**a**) Deformation behaviour of the canal levees has been analysed based on data acquired by TerraSAR-X, descending orbit (2009–2015) and visualized in the deformation velocity [mm/year] map. **(b)** A part of the levee segment in the monitored area. **(c)** A comparison is given between time-series of observed deformation *d*(*t*) and estimated deformation, $\hat{d}(t)$ using the steady-state model with an MSE of 4 mm^2^ and the *vPT*-model with an MSE of 2.1 mm^2^ for this specific PS point. The period of summer 2011 is shown in a dashed rectangle. **(d)** Cumulative precipitation [mm] and average temperature [°C] data used in the *vPT*-model. The figure was generated using the QGIS software, (version 2.18.27, https://qgis.org). The background image from Map data ©2015 Google is added using the QGIS QuickMapServices (version 0.19.10.1, https://plugins.qgis.org/plugins/quick_map_services/).

In order to analyse the deformation behaviour of the canal levees, we used 168 images recorded from the TerraSAR-X satellite, covering the case study area (see Methods for the details). We first estimate the deformation time-series of each measurement point (hereafter called PS point) on the canal levees of Delft using the PS-InSAR technique^21,37^ with an approach based on geodetic estimation theory^38^. Deformation time-series are created spanning a period of 6 years (2009–2015) (Fig. 1(a)). Although the results are visualized by linear deformation velocity [mm/year], every PS point has a complete time-series of deformation estimates in the Line-of-Sight (LOS) direction.

To examine the swelling and shrinkage behaviour of the levees, we developed a predictive deformation model, called *vPT*-model (see Methods for the details), considering the meteorological loading conditions (i.e., precipitation and temperature) as indirect indicators of the water content inside the levee. The model is used to analyse the swelling and shrinkage behaviour, and to assess whether these sub-seasonal patterns can be identified in the deformation time-series. Figure 1(b) shows a part of the levee segment in the monitored area, and an associated time-series. A comparison between the time-series of observed deformation from satellite, *d*(*t*) and the deformation $\hat{d}(t)$ estimated using the *vPT*-model, is given in Fig. 1(c) for a random PS point and compared with the steady-state model. It can be seen how the steady-state model only describes the interannual subsidence trend, whereas the *vPT*-model, which uses the meteorological data shown in Fig. 1(d), also follows the sub-seasonal swelling-shrinkage variations of the deformation time-series.

Nevertheless, deviations from the *vPT*-model occur, e.g., in December 2010 and in summer 2011, see Fig. 1(c). For example, the summer period of 2011, indicated by a dashed rectangle in Fig. 1(c), was very dry. During this period, surface of the levees was sprayed with water in order to avoid excessive drying of the soil due to the extreme drought conditions. This situation may explain the unexpected deformation of the levee which showed a swelling behaviour not predicted by the model.

### Application of the *vPT* deformation model

**Figure 2:**
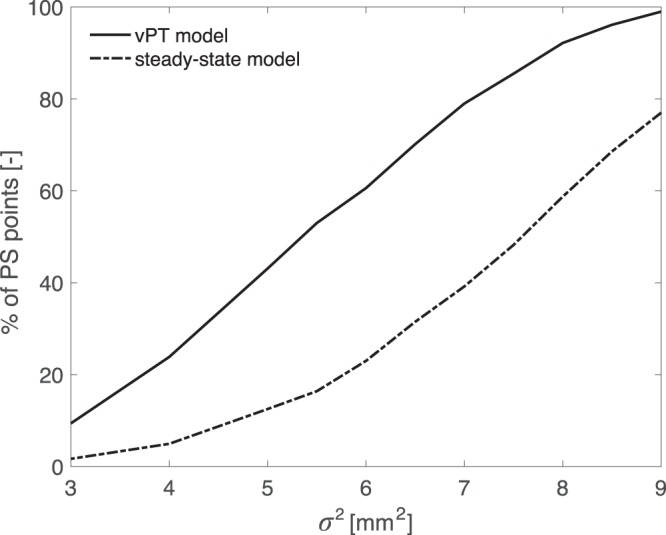
Overall Model Test (OMT) results. Percentage of PS points for which *vPT*-model and steady-state model are sustained for different values of variance of unit weight, *σ*^2^.

The developed *vPT*-model has been applied on each PS point along the canal levees in Delft, comprising of 1184 PS points. To assess how well the *vPT*-model describes the deformation compared to the steady-state model, we apply hypothesis testing to both models independently. Firstly, the significance of the *vPT*-model is tested using an Overall Model Test (OMT) (see Methods, for the details) and compared with the steady-state model for different values of the assumed variance of the observations, ${\sigma }_{d}^{2}$. A lower value for ${\sigma }_{d}^{2}$ increases the value of the test statistic and thus the probability that the null hypothesis, *H*_0_, is rejected. The results are given in Fig. 2, showing the percentage of PS points for which *H*_0_ is sustained. It can be seen that, already for ${\sigma }_{d}^{2}=9\,{{\rm{mm}}}^{2}$, the *vPT*-model is providing a higher number of PS time-series that can be well modelled compared to the steady-state model. By decreasing the value of ${\sigma }_{d}^{2}$, the difference in significance between the two models gets even larger. This higher significance is the result of the improved modelling capability of the *vPT*-model, which results in better estimates of the observed deformation time-series.

**Figure 3:**
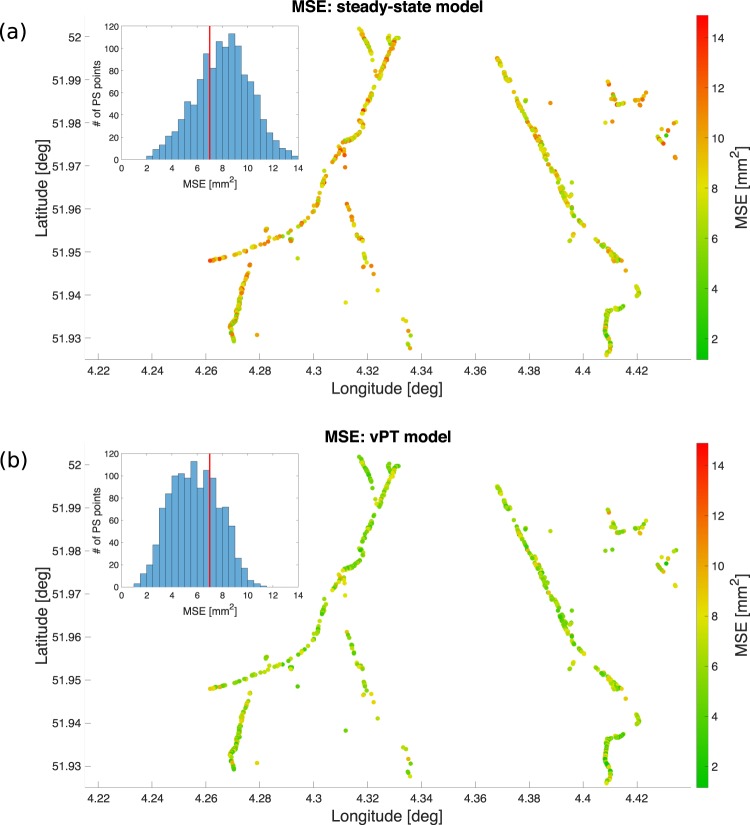
Modelling results for the deformation behavior of the canal levees in Delft, the Netherlands, cf. Fig. 1. MSE values per PS point and distribution of MSE are shown for **(a)** steady-state model, **(b)** *vPT*-model. The comparison between the two maps shows how the *vPT*-model generally provides a lower MSE over the entire levee structure.

In order to quantify this improved modelling capability, we then evaluate the quality of the estimations by calculating the mean square error (MSE) for each PS time-series. In Fig. 3, the MSE value per PS point for the entire area is given for both steady-state and *vPT*-model. The reduction in the MSE for the *vPT*-model compared to the steady-state model can be clearly observed on the two maps, with green points representing the PS time-series showing a low MSE, and red points representing PS time-series giving high MSE. The MSE values are also given in the histograms, which illustrates the error distribution for the PS points considered. The comparison between the two distributions highlights a clear shift of the MSE towards lower values.

The reduction in the MSE also allows to assume a lower variance ${\sigma }_{d}^{2}$ for the observations. For instance, assuming an a-priori variance of 7 mm^2^, the *vPT*-model gives 79% of the PS points with ${\rm{MSE}} < {\sigma }_{d}^{2}$, while the steady-state model gives only 39%. Hence, for a large number of points, the deformation data can be modelled using the *vPT*-model with higher quality of the estimations. Thus, for those points that have low MSE, the *vPT*-model can also provide better indications of the swelling and shrinkage patterns.

However, deformation estimations with low precision (e.g. MSE above 7 mm^2^) are still included in the results. In general, a high MSE could be related to several factors, such as radar signal decorrelation^20,38^ (e.g., vegetation, maintenance), or unmodelled deformation behaviour due for instance to problematic locations or soil compositions responding differently to meteorological changes^4^.

### Relation with soil types of the levee

**Figure 4:**
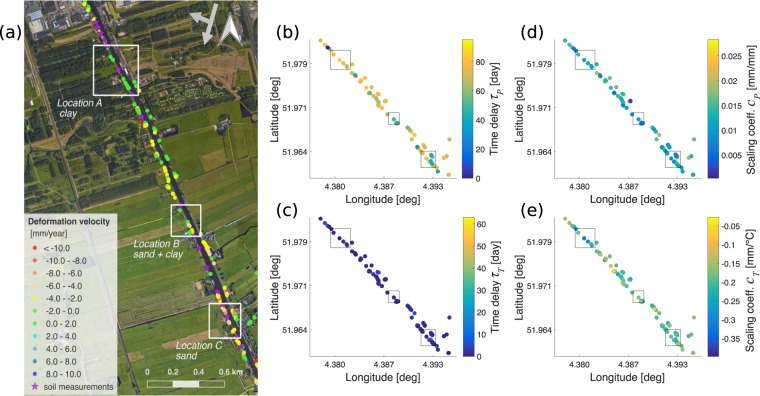
The relation between the soil types and the *vPT*-model parameters. The analysis of the estimated *vPT*-model parameters for those PS point with MSE lower than 7 mm^2^ within **(a)** the selected locations A, B and C (indicated by the rectangles) on the east levee in the study area with their predominant soil types. **(b)** time delay for *P*(*t*), *τ*_*P*_ [day], **(c)** time delay for *T*(*t*), *τ*_*T*_ [day], **(d)** scaling coefficient for *P*(*t*), *c*_*P*_ [mm/mm], **(e)** scaling coefficient for *T*(*t*), *c*_*T*_ [mm/°C]. The figure in (a) was generated using the QGIS software, (version 2.18.27, https://qgis.org). The background image from Map data ©2015 Google is added using the QGIS QuickMapServices (version 0.19.10.1, https://plugins.qgis.org/plugins/quick_map_services/).

The deformation behaviour of a levee depends on its soil characteristics. In order to assess whether any deformation pattern related to the type of soil can be observed from the model parameters, we focus on a levee segment (Fig. 4(a)) for which the soil profiles are provided by the local water authority, Water Board Delfland. Given these soil profiles, we defined three specific levee locations given in Fig. 4, whose main soil type for the first 2 meters of depth are considered. Location A is predominantly made of clay, location B shows different mixtures of clay and sand, and location C has sand as its primary soil type. These three locations have been investigated further based on the *vPT*-model results for those PS points giving an MSE lower than 7 mm^2^. First, we compared the reaction time of the soil, i.e. time delay parameters *τ*_*P*_ and *τ*_*T*_. Then, we analysed the scaling coefficients *c*_*P*_ and *c*_*T*_, which are expected to provide an indication about the reaction magnitude of the different soil types.

Considering the swelling-shrinkage behaviour of the types of soil, sandy soil is expected to react faster than clayey soil for the same amount of precipitation received due to larger porosity and higher hydraulic conductivity. On the other hand, clayey soil would react with bigger magnitude compared to the sandy soil due to the organic components in its composition. Large pore volumes in between sand particles would allow the water to drain quicker, which would result in smaller volume changes compared to clayey soil.

In order to verify if these expected behaviours are observed in the parameters of the estimated *vPT*-model, Fig. 4(a) shows the different PS points within the selected locations (indicated by the rectangles), where the estimated values of the *vPT*-model parameters are given on a colour scale in Fig. 4(b–e). Figure 4(b) shows the values for the time delay *τ*_*P*_ related to the precipitation data. Location A shows longer *τ*_*P*_ compared to locations B and C. This is in accordance with the expected faster reaction of sandy soil compared to clayey soil. On the other hand, the values of the *τ*_*T*_ parameter (Fig. 4(c)) are almost always zero. This is an indication that the levee reacts almost instantaneously with respect to temperature (averaged over 10 days) regardless of the soil type. The few PS points showing higher values for *τ*_*T*_ are considered to be outliers, for which the parameters might have been poorly estimated due to some of the issues discussed before.

The values of the scaling coefficient related to the precipitation data *c*_*P*_ provide no clear indication about the reaction magnitude of the different soil types. A better idea is however provided by the scaling coefficient for the temperature *c*_*T*_ in Fig. 4(e). In this case, it is more evident that the influence of the temperature on the clayey soil (location A) is stronger than on sandy soil (location C).

In general, these results are in accordance with the expected behaviour of different soil types to precipitation and temperature changes, regardless of the fact that only a small number of PS points with low MSE is available in each location considered. The variability of the parameter values within the same locations is most likely due to differences in levee characteristics (e.g. different slopes), and heterogeneity of the soil profiles in between two boring measurement locations and other factors. More quantitative results and a better validation of the *vPT*-model for different soil types would thus require a larger amount of data available and of better quality, and more detailed information about the soil type at specific locations.

## Enhancing Flood Protection

The strength of the proposed *vPT*-model lies in the ability of describing not only the interannual subsidence phenomenon, but also the sub-seasonal deformation behaviour using meteorological data, i.e., precipitation and temperature, as indirect indicators of the water content inside the levee. This also allows analysing the influence of the meteorological conditions on the swelling and shrinkage behaviour on a weekly basis, thus enabling predictions of the expected behaviour of the levee due to variations in the loading conditions. Being able to model and predict the sub-seasonal behaviour of a levee based on recorded extreme meteorological events would thus increase our ability of identifying critical situations.

In addition, the observed deformation can also be related to changes in geohydrological properties of a levee, such as the moisture content, weight and phreatic line (i.e. groundwater table in an unconfined aquifer). An instability or sliding of the landside slope can in fact occur when the levee loses weight due to drying and/or due to changes in effective stress in the soil. In order to illustrate the potential of satellite monitoring to detect stability problems, we consider the canal levee failure that occurred near Wilnis, the Netherlands, in 2003, as an example. Extreme drying out of this peat levee during the hot summer led to its loss of weight, triggering levee instability which consequently lead to flooding of the neighbourhood behind it^39^. Using the documented information by Van Baars^39^ on the weight changes from saturated to unsaturated conditions of the levee and assuming isotropic deformation, we roughly estimate 2 to 10% of shrinkage on the crest before the actual failure. In case of a deformation mostly on the vertical direction, this range can increase up to 20% during a dry period of 100 days (see Methods for the details). Expected deformations in the first phases of the drying process are estimated to be already in the range of few centimetres. While no SAR satellite data were available in the period of failure, this range of deformation is well within the observability capabilities of current SAR sensors and PS-InSAR algorithms. The monitoring and modelling of this kind of deformation behaviour, which normally takes place over a period of several weeks to months and eventually leads to a failure, could help to flag the extreme shrinkage of the levee. This would allow levee managers to apply timely countermeasures, such as watering or installing stability berms, to prevent instability.

A related possible future application of the proposed *vPT*-model is the detection of deformation anomalies in the framework of an early warning system. For example, when the soil has dried, the shrinkage and the volume change may result in cracks in the soil. In this case, the deformation behaviour of the levee will deviate from the expected deformation. This particular event would then be regarded as an anomaly with respect to the normal behaviour of the levee, which should be an indication for a potential weakness and a call for more in-depth analysis of the situation.

It is noted that levee deformation and its effect on safety will be highly dependent on local soil and geohydrological conditions of the levee. Further research on these aspects would need to address the relationship between deformation and geohydrological levee properties and the effects on levee stability for a number of representative situations.

In conclusion, this study shows that (a) sub-seasonal deformations obtained from monitoring a levee with the PS-InSAR technique can be observed on the time scale of weeks, that (b) these deformations are strongly correlated with the changes in meteorological conditions, that (c) deformation changes in time can be estimated using a relatively simple regression model, and that (d) deformations can be directly related to geohydrological properties and the safety of the levee. Even though the examples are given for the Netherlands, this technology is applicable to other parts of the world, thus supporting levee management especially in countries with extensive flood defence systems. Findings of this study will assist the future development of reliable early warning methods using continuous deformation monitoring, thus enhancing flood protection.

## Methods

### Study area

This paper focuses on a 10 × 10 km area south of Delft, the Netherlands. The levees used in this study are regional flood defences, as they are situated along regional rivers and canals. The canals are used to drain excess water from the lower-lying polders to the main rivers and the sea.

### Meteorological data

Precipitation [mm/day] and temperature [°C] data are obtained from meteorological station 344 from the Royal Dutch Meteorological Institute near Rotterdam. Both precipitation and temperature are measured hourly with electronic sensors with a precision of 0.1 mm and 0.1 °C, respectively. The distance from the meteorological station to the study area is approximately 4 km. Hence, the measured data are expected to differ slightly from the meteorological conditions at the study area. However, since cumulative and average meteorological values are used in our model, the effect is assumed to be negligible.

### Soil data

Soil profiles were obtained from borings performed in 2011 by Water Board Delfland at 17 different locations along the levee. Taking into account the non-uniformity of the soil compositions and the changes in soil moisture content of unsaturated zone, the dominant soil type from 0 to 2 meters below the surface level is being considered.

### Deformation data from PS-InSAR processing

The case study area has been monitored using data from TerraSAR-X to estimate the deformation time-series of each PS point between 8 April 2009 and 8 January 2015. This satellite provides X-band high resolution data with a wavelength of 31 mm, 3 × 3 m pixel size and a repeat cycle of 11 days.

The main principle of satellite radar imaging can be described as follows. Radar sensors transmit pulses of high-frequency electromagnetic waves from space to Earth and record the strength and the fractional phase of the back-scattered signals that are reflected from the surface to construct SAR images. By interfering at least two radar images acquired at different times over the same location, the combined effect of surface deformation, topography and atmospheric signal delay is obtained. In order to estimate and isolate the surface deformation from the other phase contributions, a large stack of SAR images acquired by the same satellite is analysed by interferometric time-series methods. All deformations are projections of the real deformation [mm] onto the Line-of-Sight (LOS) direction. This direction from satellite to object is determined by the heading angle of the satellite, *α*_*h*_, and the incidence angle of the radar, *θ*_*inc*_.

Various time-series processing techniques can be applied to estimate the deformations from the satellite data. The most suitable approach depends on a number of factors, such as the number of available radar images, satellite characteristics, the area of interest (e.g., surface cover), and the expected deformation signal. Regardless of the specific approach used, PS-InSAR analysis typically includes three main steps; 1) stack processing: creating the multiple interferograms from complex data, 2) PSI analysis: detecting Persistent Scatterers (PS) and separating deformation phase from other contributions (such as topography, atmospheric delay^20^) and 3) quality assessment: evaluating the quality of the results^38^. In this study, the interferometric stack processing of the radar data has been performed using the Delft Object-oriented Radar Interferometric Software (DORIS)^40^. The Delft implementation of Persistent Scatterer InSAR (DePSI)^38^ has been applied on 168 TerraSAR-X strip-map images in order to estimate the Line-of-Sight (LOS) deformation time-series. The main principles of PS-InSAR and a general overview of the past studies can be found in a review^41^, whereas another study^4^ discusses the applicability of the technique to continuous levee monitoring.

### Deformation Modelling

In order to describe this deformation behaviour of earth-filled levees, we consider its relation with respect to those meteorological data, i.e. precipitation and temperature, which are expected to give an indication of soil moisture changes. For this reason, the steady-state model, which considers the interannual trend, due to the long-term irreversible behaviour of the levee (e.g. subsidence), is extended with the introduction of precipitation, *P*, and temperature, *T*, time-series. In this way, it is also possible to evaluate the sub-seasonal and reversible behaviour of the levee, i.e. its swelling and shrinkage. Hence, the proposed model, hereafter called *vPT*-model, is defined as

1 $$d(t)={d}_{{\rm{V}}}(t)+{d}_{{\rm{PT}}}(t),$$

where the first term corresponds to the steady-state model,

2 $${d}_{{\rm{V}}}(t)=v\cdot t+b,$$

with *v* the slope in [mm/day] and *b* the intercept in [mm] of the long-term linear trend. This intercept accounts for the atmospheric signal delay and scattering noise in the master acquisition, which is common in all single-master interferograms^38^. The second term describes the swelling-shrinkage behaviour of the levee as a linear combination of precipitation and temperature time-series. We expect the soil to react to variations in precipitation and temperature only after a certain period of time. This requires a regression model which includes a time delay *τ* between the meteorological data and the observed levee deformation. The second term of equation (1) is then defined as

3 $${d}_{{\rm{PT}}}(t)={c}_{P}(P(t-{\tau }_{P})-{\delta }_{P})+{c}_{T}(T(t-{\tau }_{T})-{\delta }_{T}),$$

where the time-series at time *t* are indicated as *d*(*t*) in [mm] for deformation, *P*(*t*) for the cumulative precipitation, in [mm], over a time interval Δ*t*_*P*_, starting at *t* − Δ*t*_*P*_ and ending at *t*, and *T*(*t*) for the average temperature, in [°C], over a time interval Δ*t*_*T*_, starting at *t* − Δ*t*_*T*_ and ending at *t*. The offsets for precipitation and temperature time-series are represented by *δ*_*P*_ in [mm] and *δ*_*T*_ in [°C], respectively, while the time delay parameters for *P*(*t*) and *T*(*t*) with respect to *d*(*t*) are denoted as *τ*_*P*_ and *τ*_*T*_, with their units in [day]. Lastly, *c*_*P*_ in [mm/mm] and *c*_*T*_ in [mm/°C] are the scaling coefficients of the linear combination, between *d*(*t*) and *P*(*t*) and between *d*(*t*) and *T*(*t*), respectively.

### Model parameter estimation

Soil deformation is expected to result from cumulative and smooth variations in precipitation and temperature. For this reason, the mean temperature and the cumulative precipitation data over time periods Δ*t*_*T*_ = 10 days and Δ*t*_*P*_ = 30 days are considered, respectively. The time period for the cumulative precipitation was chosen to be longer than the time resolution of the deformation data to take into account for the long-term effect of precipitation.

The *vPT*-model in equation (1) can be simplified as

4 $$d(t)=v\cdot t+{c}_{P}(P(t-{\tau }_{P}))+{c}_{T}(T(t-{\tau }_{T}))-\delta $$

where *d*(*t*), *P*(*t*) and *T*(*t*) are the deformation and meteorological data preprocessed as described above, and the global offset coefficient is defined as *δ* = (*c*_*P*_*δ*_*P*_ + *c*_*T*_*δ*_*T*_ − *b*). This model is non-linear due to the products *c*_*P*_*τ*_*P*_ and *c*_*T*_*τ*_*T*_. For this reason, we use the cross-correlation method^42^ to estimate the *τ*_*P*_ and *τ*_*T*_ parameters. This approach is used to shift *P*(*t*) and *T*(*t*) with respect to *d*(*t*) (after removing the steady-state trend *v*⋅*t*) and to compare the two records at each possible time delay, where ${\hat{\tau }}_{P}$ and ${\hat{\tau }}_{T}$ are selected as the values providing the maximum absolute value in the cross-correlation function. Hence, the precipitation and temperature time-series are aligned to the deformation data (i.e. shifted by ${\hat{\tau }}_{T}$ and ${\hat{\tau }}_{P}$, respectively).

After the time alignment, the *vPT*-model is simplified as

5 $$d(t)=v\cdot t+{c}_{P}\tilde{P}(t)+{c}_{T}\tilde{T}(t)+\delta ,$$

where the aligned time-series are defined by

6 $$\tilde{P}(t)=P(t-{\hat{\tau }}_{P}),\,\,\tilde{T}(t)=T(t-{\hat{\tau }}_{T}).$$

The optimal values for the linear parameters of the *vPT*-model are estimated by minimizing the mean square error between the deformation data and the model estimate

7 $$\mathop{min}\limits_{x}||{\boldsymbol{d}}-{\boldsymbol{Ax}}{||}_{2}^{2},$$

8 $${\boldsymbol{x}}=[v,{c}_{P},{c}_{T},\delta ]^{\prime} ,\,{\boldsymbol{A}}=[{\boldsymbol{t}},\tilde{{\boldsymbol{P}}},\tilde{{\boldsymbol{T}}},{\bf{1}}]^{\prime} $$

where ***d*** is the *m* × 1 vector containing the LOS deformation observations, ***A*** is the *m* × *n* design matrix whose columns are the time vector ***t***, the vectors $\tilde{{\boldsymbol{P}}}$ and $\tilde{{\boldsymbol{T}}}$ containing respectively the aligned precipitation $\tilde{P}$ and temperature data $\tilde{T}$, **1** is a vector containing only ones, and ***x*** is the *n* × 1 vector of the model parameters (the symbol ′ indicates the transpose). The estimated optimal values of ***x*** are then given by the least squares solution^43^ as

9 $$\hat{{\boldsymbol{x}}}={({\boldsymbol{A}}{\boldsymbol{^{\prime} }}{{\boldsymbol{Q}}}_{d}^{-1}{\boldsymbol{A}})}^{-1}{\boldsymbol{A}}{\boldsymbol{^{\prime} }}{{\boldsymbol{Q}}}_{d}^{-1}{\boldsymbol{d}},$$

where the covariance matrix ***Q***_*d*_ specifies the dispersion of the measured deformation data. The observations in the time-series are assumed to be uncorrelated, each having a fixed variance of unit weight ${\sigma }_{d}^{2}$. Thus, the variance matrix can be factorized as ${{\boldsymbol{Q}}}_{d}={\sigma }_{d}^{2}{{\boldsymbol{I}}}_{m}$, with ***I***_*m*_ an *m* × *m* identity matrix.

Once the model parameters are obtained as explained above, they are used in the *vPT*-model of equation (4) to obtain the estimated (adjusted) deformation time-series $\hat{{\boldsymbol{d}}}={\boldsymbol{A}}\hat{{\boldsymbol{x}}}$. The error between the measured deformation time-series and the estimated one is given by the residual $\hat{{\boldsymbol{e}}}={\boldsymbol{d}}-\hat{{\boldsymbol{d}}}$ and the quality of the estimation is then evaluated by the mean square error (MSE).

### Hypothesis testing: Overall Model Test (OMT)

The Overall Model Test (OMT)^44^ is used to check the validity of the models. Testing is usually performed by comparing a *null hypothesis*, *H*_0_, versus an *alternative hypothesis*, *H*_*a*_, where *H*_0_ represents the model under investigation and *H*_*a*_ corresponds to the case where no restrictions are imposed on the observations, as in

10 $$\begin{array}{cc}{H}_{0}:E\{{\boldsymbol{d}}\}={\boldsymbol{Ax}}, & D\{{\boldsymbol{d}}\}={{\boldsymbol{Q}}}_{d}\\ {H}_{a}:E\{{\boldsymbol{d}}\}\in {{\mathbb{R}}}^{m}, & D\{{\boldsymbol{d}}\}={{\boldsymbol{Q}}}_{d},\end{array}$$

where *E*{⋅} and *D*{⋅} denote expectation and dispersion, respectively. To reject or sustain *H*_0_ depends on the test statistic,

11 $${T}_{q}={{\hat{{\boldsymbol{e}}}}^{{\rm{^{\prime} }}}}_{0}{{\boldsymbol{Q}}}_{d}^{-1}{\hat{{\boldsymbol{e}}}}_{0},$$

which follows a central *χ*^2^-distribution with *q* = *m* − *n* degrees of freedom, and corresponds to a weighted sum-of-squares of the least squares residual vector ${\hat{{\boldsymbol{e}}}}_{0}$ under *H*_0_. Given a chosen level of significance *α*, the critical value *k*_*α*_ follows from the *χ*^2^-distribution to test the null hypothesis,

12 $${\rm{reject}}\,{H}_{0}\,{\rm{if}}\,{T}_{q} > {k}_{\alpha }\mathrm{.}$$

Rejection of *H*_0_ indicates that the deformation behaviour is not significantly well described by the chosen model, given the assumed level of significance. For InSAR time-series, *α* is typically defined in the range of 0.2% < *α* < 2%, as we prefer to stick to a relatively simple null hypothesis if possible^45^. In this study, we assumed an *α* value of 1%, but in practice this is a decision to be taken by the local authorities. The variance of unit weight for TerraSAR-X with a 31 mm wavelength is conservatively assumed to be ${\sigma }_{d}^{2}={3}^{2}\,{{\rm{mm}}}^{2}$ ^38,45^.

### Deformation estimation for the levee failure at Wilnis, the Netherlands

In the assessment of the failed levee at Wilnis^39^, two scenarios for changes of the phreatic line in the peat levee are considered: (a) fully saturated (phreatic line is at the crest), and (b) unsaturated (phreatic line drops 1 m below the crest level). Relevant information and the cross section of the levee can be found in the original study^39^. Here we estimate the expected deformation of the crest in case the phreatic line drops from scenario (a) to scenario (b), using the data documented by Van Baars^39^. In scenario (a), the volume of saturated soil, *V*_soil-sat_ = 1 m^3^. The gravimetric water content, Θ, is described as the ratio between the mass of the water, *M*_*w*_, and the mass of solids, *M*_solids_. For the saturated peat soil at the Wilnis levee, Θ was varying between 600% to 800%, and the unit weight of the saturated peat soil, *γ*_*sat*_ = 11 kN/m^3^, and the unit weight of unsaturated peat soil, *γ*_unsat_ = 5 kN/m^3^ ^39,46,47^. The ratio between mass and volume of the solids is assumed to be constant.

In the case of fully saturated soil (scenario (a)), the mass of soil, *M*_soil-sat_, is equal to sum of mass of water, *M*_w-sat_ and mass of solids, *M*_solid-sat_. Thus, for the given Θ of the saturated soil, *M*_soil-sat_ = *γ*_*sat*_.*V*_soil-sat_ = 11 kN. Using an average Θ of 700%, *M*_*w*-*sat*_ = 9.63 kN and *M*_solid-sat_ = 1.38 kN. Considering the unit weight of water, *γ*_*w*_ = 9.81 kN/m^3^, the volume of water in the saturated soil is calculated as *V*_w-sat_ = *M*_w-sat_/*γ*_w_ = 0.98 m^3^ and the volume of solids in the saturated soil is then *V*_solid-sat_ = *V*_soil-sat_ − *V*_w-sat_ = 0.02 m^3^.

After a dry period of approximately 100 days, soil samples taken from the crest of the levee show that Θ of the unsaturated soil (scenario (b)) was around 200%^39,46^. In this case, the mass of water, *M*_w-unsat_ reduces to 2.75 kN and the corresponding volume of water in the unsaturated soil is *V*_w-unsat_ = 0.28 m^3^. The mass of solids in the unsaturated soil remains unchanged, *M*_solid-unsat_ = 1.38 kN.

Hence, the volume change of the soil, ΔV, between the two scenarios is estimated as 0.175 m^3^ in the case of a 1 m drop in the phreatic line with the reduction of Θ from 700% to 200%. This correspond to a deformation of 6% of the total height (i.e., 6 cm) assuming an isotropic shrinkage of the soil. For an initial Θ of 800% and 600%, about 2% to 10% of shrinkage can be observed, respectively. In case of an anisotropic deformation, mostly on the vertical direction, this range can increase up to approximately 20% in a drought period of 100 days.
